# Secondary polycythemia as a rare cause of ischemic stroke: A case report

**DOI:** 10.1016/j.radcr.2026.06.067

**Published:** 2026-07-09

**Authors:** Ferdaoues Ben Chihi, Meriam Messelmani, Hela Ghedira, Nejiba Fekih Mrissa, Jamel Zaouali

**Affiliations:** a Faculty of Medicine of Tunis, University Tunis El Manar, Tunis, Tunisia; b Department of Neurology, Military Hospital of Tunis, Tunis, Tunisia; c Department of Hematology, Military Hospital of Tunis, Tunis, Tunisia; d Molecular Biology Unit (UR17DN06), Laboratory of Hematology, Military Hospital, Tunis, Tunisia

**Keywords:** Stroke, Hematologic causes, Polycythemia, Secondary polycythemia, Chronic hypoxia, Risk factors

## Abstract

Polycythemia is a known but underrecognized risk factor for ischemic stroke, particularly in the setting of chronic hypoxia. We report a rare case of pontine infarction associated with secondary polycythemia in a patient with chronic respiratory failure. A 52-year-old middle-aged man with a history of hypertension, combined pulmonary fibrosis and emphysema syndrome, receiving long-term oxygen therapy as well as bronchodilator treatment, presented with sudden-onset left-sided hemiparesis. Neurological examination confirmed a pyramidal syndrome. Brain MRI revealed an acute infarct in the left pons. Laboratory findings showed a hematocrit of 62.5% and elevated hemoglobin (20.1 g/dL). Work-up excluded primary polycythemia and revealed an elevated erythropoietin level (78.2 mIU/mL), consistent with a secondary hypoxia-induced cause. Oxygen saturation on room air was 88%. No atherosclerotic or cardioembolic sources were found. The patient underwent 6 therapeutic phlebotomy sessions over 3 weeks, which reduced hematocrit to 48.7%. Neurological symptoms improved within 4 days, with a modified Rankin Score improving from 3 to 1 at discharge. This case highlights the importance of considering secondary polycythemia in patients presenting with cryptogenic stroke and underlying chronic hypoxia. Early diagnosis and targeted interventions, including phlebotomy and antiplatelet therapy, can prevent recurrence and improve outcomes.

## Introduction

Cerebrovascular events, or strokes, are the second leading cause of death worldwide, affecting over 16 million people annually [1]. While stroke can occur at any age, the underlying causes in middle-aged or younger patients often differ from those in older adults, particularly in terms of risk factors and pathophysiology. In otherwise healthy individuals, strokes may result from less common etiologies such as patent foramen ovale, vasculitis, coagulopathies, genetic disorders, or hematologic abnormalities.

Although these nonatherosclerotic causes account for a smaller proportion of cases compared to atherosclerotic causes of ischemic stroke, they are increasingly recognized in clinical practice and warrant careful evaluation. Among hematologic disorders, polycythemia—whether primary (polycythemia vera) or secondary—has been associated with an increased risk of focal cerebral ischemia due to hyperviscosity and thrombosis [2].

In this report, we present a case of brainstem infarction in a patient with a hypercoagulable state related to secondary polycythemia induced by chronic hypoxia, highlighting the diagnostic and therapeutic challenges involved.

## Case presentation

A 52-year-old man with a history of arterial hypertension and chronic respiratory failure due to combined pulmonary fibrosis and emphysema (CPFE) syndrome, on home oxygen therapy for 1 year, presented to the Emergency Department of the Military Hospital of Tunis, Tunisia with sudden-onset right brachial monoparesis, faciobrachial paresthesia, and slurred speech lasting for 2 days. He had a 52 pack-year smoking history and a previous ischemic stroke in 2022, for which he had been prescribed aspirin but discontinued it on his own.

The patient was admitted to the Department of Neurology of the Military Hospital of Tunis, Tunisia.

On neurological examination, the patient had dysarthria, right brachial monoparesis, and cerebellar ataxia. The National Institutes of Health Stroke Scale (NIHSS) score was 5. The modified Rankin Score (mRS) was 3 at admission.

## Diagnostic studies

A non-contrast brain CT revealed sequelae of a prior right superficial middle cerebral artery infarct and spontaneous hyperdensity of intracranial vessels (Fig. 1). Brain MRI with MR angiography showed an acute left paramedian pontine infarct without evidence of atherosclerosis (Fig. 2). Echo-Doppler of the supra-aortic trunks revealed a dominant right vertebral artery.

**Fig. 1:**
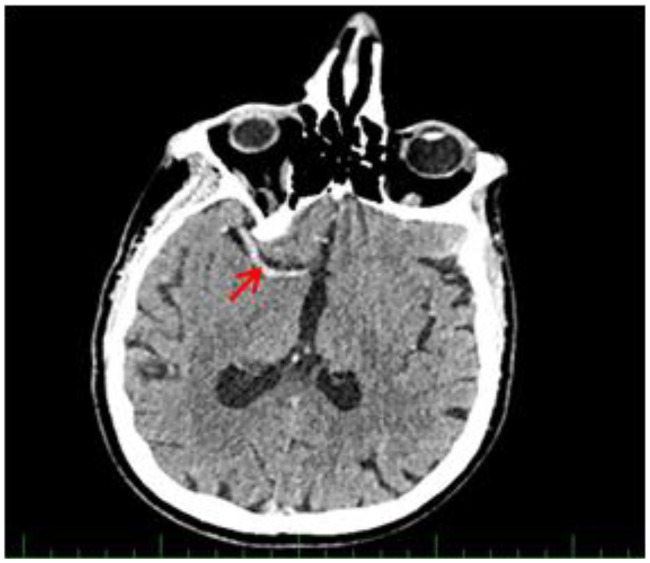
Noncontrast CT scan of the brain showing spontaneous hyperdensity in the right MCA (red arrow), indicative of thrombosis or slow flow.

**Fig. 2:**
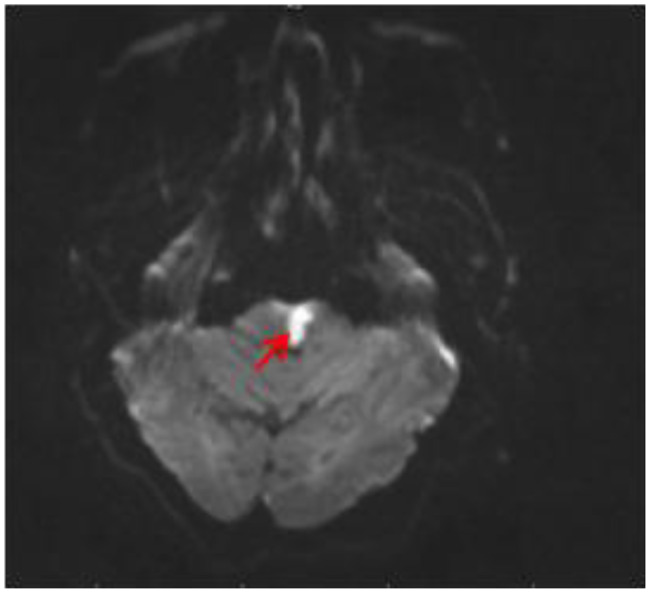
Brain MRI diffusion-weighted imaging (DWI) demonstrating an acute left paramedian pontine infarct (white arrow), corresponding to the patient’s neurological deficits.

Laboratory investigations confirmed polycythemia: red blood cell count was 6.99 million/µL (reference: 4.00-5.70), hemoglobin 20.8 g/dL (reference: 13.5-17.0), and hematocrit 62.5% (reference: 37.0-50.0). White blood cell count was 13,800/µL, and platelet count was 263,000/µL. Peripheral blood smear was normal. His arterial blood gas analysis showed PaO_2_: 56 mmHg, PaCO_2_: 49 mmHg, and pH: 7.38, consistent with chronic compensated respiratory failure. Vitamin B12 was 258 pg/mL, and homocysteine was elevated at 22.58 µmol/L. Testing for the JAK2V617F mutation, thrombophilia screening, and autoimmune markers was negative, except for a positive rheumatoid factor.

Transthoracic echocardiography and troponin levels showed no embolic source. C-reactive protein (CRP) was within normal limits. Serum erythropoietin was elevated (78.2 mIU/mL), supporting a diagnosis of secondary polycythemia due to chronic hypoxia.

A noncontrast CT scan showed sequelae of right superficial middle cerebral artery infarct and spontaneous hyperdense intracranial vessels (Fig. 1). Brain magnetic resonance imaging (MRI) with angio MR revealed an acute left paramedian pontine infarct without atherosclerosis (Fig. 2). The supra-aortic trunk echo-Doppler showed a dominant right vertebral artery. Lab tests confirmed polycythemia: His red blood cells were 6.99 m/cumm (normal range: 4.00-5.70 m/cumm), hemoglobin was 20.8 g/dL (normal range: 13.5-17.0 g/dL), and hematocrit was 62.5% (normal range: 37.0%-50.0%), white cell count was 13,800 and thrombocyte count was 263,000. Blood film was normal. Vitamin B12 (258 pg/mL) and homocysteine (22.58 µmol/L) were measured. JAK2V617F mutation, thrombophilia screening, and autoimmune markers were negative, except for rheumatoid factor positivity.

Transthoracic echocardiogram and troponin showed no embolic source. His C-reactive protein (CRP) level was normal. The final diagnosis was multiple thromboembolic strokes due to secondary polycythemia from chronic hypoxia.

## Therapeutic intervention and outcomes

The patient was started on aspirin (100 mg/d) and atorvastatin (40 mg/d). Hematology consultation recommended therapeutic phlebotomy. He underwent 6 phlebotomy sessions, each removing 200 mL of blood, performed every other day over a 2-week period. This reduced his hematocrit to 48.7%. Neurological symptoms improved significantly within 4 days of initiating phlebotomy and supportive care. During hospitalization, he developed an acute exacerbation of COPD complicated by heart failure, which was managed successfully. At discharge, the modified Rankin Score had improved to 1. He was discharged on aspirin, statins, and maintenance therapy for CPFE syndrome and heart failure.

## Discussion

Very few cases of thrombotic events associated with polycythemia, especially secondary to chronic hypoxemia, have been reported in the literature [[2], [3], [4], [5]]. Polycythemia refers to an increased red blood cell mass and is classified into primary and secondary forms. In 2016, the World Health Organization (WHO) redefined polycythemia vera—a myeloproliferative neoplasm—as hemoglobin >16.5 g/dL and/or hematocrit >49% in males, and hemoglobin >16.0 g/dL and/or hematocrit >48% in females [6]. Although these thresholds were originally intended for the diagnosis of polycythemia vera, they are often used as reference values for identifying secondary polycythemia as well.

Thrombosis occurs in 20%-33% of patients with polycythemia vera, with a third experiencing recurrent events [7,8]. Although less well documented in secondary forms, several case reports have described thromboembolic complications, including ischemic strokes, in the setting of secondary erythrocytosis due to chronic hypoxia, renal tumors, or exogenous erythropoietin administration [2,4,[9], [10], [11]].

The pathophysiology of stroke in polycythemia is thought to involve hyperviscosity-induced stasis, increased platelet-endothelial interaction, and microvascular flow impairment [12]. These changes are especially critical when hematocrit exceeds 45%, with risk increasing proportionally beyond this threshold [12]. In our case, the patient presented with a hematocrit of 62.5%, well above the danger range. His neurological deficits improved markedly within 4 days after initiating therapeutic phlebotomy and supportive care, during which hematocrit was reduced to 48.7%. This temporal relationship supports the hypothesis that hyperviscosity was a key mechanism.

Chronic hypoxia remains the most frequent cause of secondary polycythemia. Common contributing conditions include COPD, interstitial lung disease, obstructive sleep apnea, and high-altitude exposure, all of which stimulate erythropoietin production and promote erythrocytosis [3,5]. In COPD patients, the prevalence of polycythemia varies between 6% and 10.2% when defined by hemoglobin ≥17 g/dL in males [13,14], and hematocrit levels ≥55% have been found in up to 8.4% of patients on long-term oxygen therapy [15].

Our patient had no evidence of atherosclerosis or cardioembolic disease. Despite cardiovascular risk factors such as smoking and hypertension, both echocardiography and vascular imaging were unremarkable. The radiological pattern was suggestive of a small-vessel or embolic stroke, but no clear source was found. In the absence of other identifiable causes, secondary polycythemia was considered the most likely contributing factor to the ischemic stroke, particularly in view of the clinical improvement following hematocrit reduction.

Phlebotomy remains the mainstay treatment in managing symptomatic or high-risk polycythemia. It has been shown to reduce blood viscosity, improve cerebral perfusion, and lower thrombotic risk [16]. In patients with polycythemia vera, low-dose aspirin (100 mg/d) has been demonstrated to be both safe and effective for preventing thrombosis [17,18]. Although no large-scale trials have been conducted in secondary polycythemia, antiplatelet therapy is considered reasonable when thrombotic risk is evident and no contraindications exist.

This case highlights the importance of recognizing secondary polycythemia as a potential cause of ischemic stroke, especially in patients with chronic hypoxic lung disease and unexplained cerebrovascular events. A thorough evaluation, including erythropoietin levels, blood gas analysis, and exclusion of alternative causes, is essential. Prompt treatment with phlebotomy and antiplatelet therapy may lead to neurological recovery and prevent future events.

To better contextualize our case, we reviewed similar reports of ischemic stroke associated with secondary polycythemia (Table 1). The reported cases illustrate diverse underlying causes, such as smoking-related polycythemia, chronic hypoxia, congenital heart disease, and paraneoplastic syndromes. Our patient shares similarities with these cases in terms of elevated hematocrit and therapeutic response to phlebotomy. However, the pontine location of the stroke and the coexistence of COPD with interstitial lung disease make our case unique. Moreover, the rapid neurological improvement observed after phlebotomy underscores the importance of prompt diagnosis and management in such patients.

**Table 1:** Summary of reported cases of ischemic stroke associated with secondary polycythemia.

Reference	Age/sex	Cause of polycythemia	Stroke location	Hematocrit (%)	Main treatment	Outcome
Thakur and Westover [2]	45/M	Smoker’s polycythemia	Cerebral infarction (unspecified)	Not specified	Antiplatelet + supportive care	Improvement after treatment
Zhou et al. [3]	50/F	Chronic hypoxia secondary polycythemia	Cerebral infarction (unspecified)	58	Phlebotomy + oxygen therapy	Partial recovery
Pandian et al. [4]	18/M	Cyanotic congenital heart disease	Acute ischemic stroke	Not specified	Phlebotomy + cardiac management	Clinical stabilization
Corse et al. [11]	60/M	Secondary polycythemia due to renal carcinoma	Left cerebral infarction	65	Phlebotomy + oncologic treatment	Improvement after hematocrit correction
Current case	52/M	Chronic hypoxia (COPD + interstitial lung disease)	Left pontine infarction	62.5	Phlebotomy (6 sessions), aspirin	Significant improvement within 4 d

## Conclusion

This case highlights the importance of considering systemic signs and uncommon causes of ischemic stroke, such as hematologic disorders, especially in patients without an obvious source of stroke. The association between elevated hematocrit levels and ischemic stroke in patients with secondary polycythemia warrants further investigation in larger cohorts to better understand its clinical impact.

## Declaration of generative AI and AI-assisted technologies in the writing process

Generative AI and AI-assisted technologies were not used in the preparation of this work.

## Patient consent

Written informed consent for publication of this case report and associated images was obtained from the patient.
